# Addressing the health of vulnerable populations: social inclusion and universal health coverage

**DOI:** 10.7189/jogh.08.020304

**Published:** 2018-12

**Authors:** Viroj Tangcharoensathien, Anne Mills, Maitreyi Bordia Das, Walaiporn Patcharanarumol, Monthian Buntan, Jeffery Johns

**Affiliations:** 1 International Health Policy Program, Ministry of Public Health, Nonthaburi, Thailand; 2 London School of Hygiene and Tropical Medicine, London, UK; 3 World Bank Group, Washington, D.C., USA; 4 National Legislative Assembly, Bangkok, Thailand; 5 Khon Kaen University, Khon Kaen Province, Thailand

**Figure:**
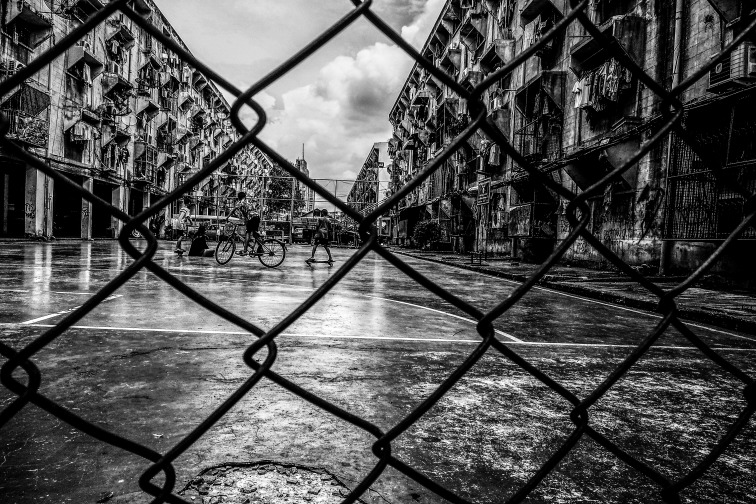
Photo: PMAC 2017 World Art Contest, Ratanan Rattana, World First Prize, Photo Category (used with permission)

The adoption of the Sustainable Development Goals (SDG) was guided by the ethical principle of leaving no one behind, which puts social justice and equity at the centre of development. Universal health coverage (UHC) is an important policy tool in achieving improved access to health services, financial risk protection and health of the population, helping ensure that indeed no-one is left behind with respect to access to and use of health services.

## THE PLIGHTS OF THE VULNERABLE POPULATION

A number of barriers exclude some individuals and groups from access to and use of health and social services and from participation in economic activities and policy spaces. Attributes such as gender, race, caste, indigenous origin, ethnicity, and religion, diseases such as HIV/AIDS and tuberculosis, and disability, migration and displacement status, are some of the axes along which people are excluded. More broadly, capability deprivation contributes to exclusion. The capability approach, introduced by Sen [1], focuses on what people are able to do and be, as opposed to what they have. This approach shifts the analytical focus from the means of living, eg, income, to the actual opportunities a person has, such as to be healthy, and their capabilities including to participate in society [2].

Social exclusion is the process of marginalizing individuals or groups of a particular society and denying them from full participation in social, economic and political activities. Social vulnerability is the inability of these individual or communities to oppose negative situations or impacts. Hence, social exclusion leads certain individuals to social vulnerability [3].

Though there are differences in perspectives, there is consensus that social exclusion is multi-dimensional, dynamic and relational. Social exclusion, characterized by unequal access to resources, capabilities and rights, is a multi-dimensional process driven by unequal power relationships across four dimensions–economic, political, social and cultural, which operates at individual, household, community, country and global levels. The relational processes, operating at macro- and micro-levels, deliberately exclude particular groups of people from engaging fully in community and social life, such as access to affordable education, and equal employment opportunities [4].

**Figure 1:**
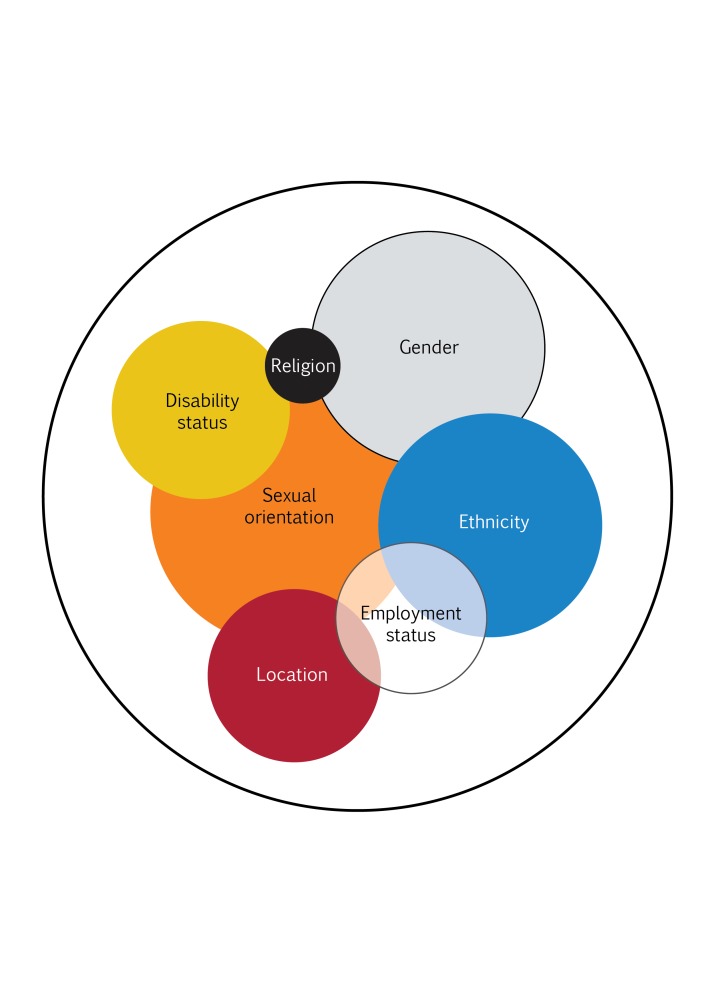
Intersectionality of different vulnerable attributes. Source [5].

The co-existence of multiple attributes multiplies disadvantage amongst already excluded groups, as seen in **Figure 1**. For example, a disabled young girl belonging to a disadvantaged caste living in a tribal area of India is categorized as the most vulnerable among the vulnerable people. This is often referred to as intersectionality [5].

The consequences of social exclusion are enormous, often making excluded groups voiceless and invisible in the society in which they live, conferring low social standing, poverty, low human capital endowments, restricted access to employment and services, and lack of social participation. In Latin America and the Carribean, for instance, persons of African descent often have lower access to markets, services and spaces. In India and Nepal, persons belonging to the lowest caste have been subjected to indignity and disrespect for centuries.

Political conflict has magnified the issue of social exclusion. Forced displacement hit a peak in 2015 even greater than that seen after World War II, with 15 million refugees, a 45% increase from 2011 that was largely due to the five-year-long armed conflicts in Syria. Alarmingly, there were five million newly displaced persons in the first half of 2015 [6]. Evidence points to the grave health consequences among refugees: physical assault, mental breakdown and depression. Refugees are more likely to be sexually abused and their human rights violated, as reported in many detention centers for asylum seekers [7]. Humanitarian actions can preserve life, relieve suffering, protect human dignity and restore people’s ability to make their own decisions. Yet, the increased volume of refugees and displaced persons far exceeds the capacity of humanitarian resources, and long term political solutions to conflicts are required.

In 2015, the number of international migrants worldwide who resided in a country other than their country of birth was the highest ever recorded, at 244 million, up from 232 million in 2013. Economic disparity across rich and poor countries, and demographic imbalances between the global north with a low fertility rate and labour shortage and the global south with labour surplus, are the main drivers of international migration.

Restrictive migration policies globally have resulted in increased vulnerability of migrants to irregularity (those who enter, stay or work in a country without necessary authorizations or documents required by immigration regulations), labour exploitation and trafficking. The 2012 economic crisis in Europe resulted in austerity measures that had negative impacts on social protection for national and migrant labour. The stereotyping and public discourse discriminating against migrants exacerbate social exclusion and xenophobia [8].

Take also the case of disability, which may have physical or mental causes, but is intensified by familial and social prejudice, stigma and discrimination. The UN Convention on the Rights of Persons with Disability (UNCRPD), ratified by 169 UN member states, called for a shift in the paradigm from medico-charity to a social model of disability which eliminates stigma and empowers independent living. There is a need for synergies with other conventions such as the Convention on Child Rights, the Convention on the Elimination of All Forms of Discrimination against Women (CEDAW), and other international human rights instruments to address the challenges of multiple vulnerabilities.

Despite the two Conventions ratified by State Parties, the UNCRPD and the CEDAW, governments still lack capacity to protect persons with multiple vulnerable attributes, such as indigenous women with disability. Forced sterilization of mentally ill persons, and of HIV positive women, is still practised [9]. A survey in 2010 by the Disabled People's International (DPI) Women's Network Japan [10] has uncovered the trauma in the lives of women with disability such as sexual abuse, forced sterilization and stigmatization at workplaces. Worldwide, 93 million children under 14 years–one out of 20 - have moderate or severe disability of some kind. Children with disability have a lower rate of primary education, 42% in girls and 51% in boys compared with 53% among non-disabled girls and 61% in boys [11].

Yet another social group is often left behind. Across Asia and Latin America, “tribal peoples” and “indigenous peoples” have lower social status, limited voice and poor health outcomes. While poverty has reduced and human development outcomes have improved across the board, yet improvements among indigenous peoples have not been as dramatic as those among the general population. The history of Stolen Generations exemplifies the abuse of ethnic minorities [12].

## POTENTIAL SOLUTIONS: CONCERTED ACTIONS

Social inclusion is defined as the “process of improving the ability, opportunity and dignity of people disadvantaged on the basis of their identity to take part in society” and should be applied in the implementation of the SDGs [13]. This requires actions by a number of actors.

Political actors have moral obligations to recognise social exclusion and strengthen capacities to identify vulnerable populations, and devise effective inter-sectoral actions to progressively realize social inclusion in markets, services, and spaces, and ensure capacities to monitor progress. These responses must be framed within a human rights framework. The state is obligated to strengthen and enforce laws and implement policies which reduce all types of stigma, discrimination and violence in all settings including employment, education and health care.

The health sector has to protect the health of the vulnerable population through progressive realization of UHC as a basic platform. Policy responses must be multisectoral - and need not be about doing more but about doing things differently [14]. Innovative interventions particularly from the demand-side such as conditional cash transfers can help, though there can be challenges in monitoring and in long-term sustainability. Strengthening the availability and functioning of health delivery systems where health workers are trained in human rights, have zero discrimination attitudes, and practise dignified services to all, should run parallel to these demand-side interventions.

Policy-makers should ensure the provision of dignified and respectful services, for example, embedding anti-stigma interventions in national HIV policy and programs. The use of a community score-cards, local assemblies, and effective dialogue between community and health care providers, can encourage “collaborative governance for health” and enhance accountability to citizens and the socially excluded.

Health professionals’ education should be transformed, in both institutional and instructional dimensions, to achieve a “socially accountable health workforce”, by providing greater opportunities for students from socially excluded groups to train as health professionals and be posted in their home communities upon graduation. This helps ensure respectful services to their local populations. Training of health workers in cultural competencies supports provision of responsive and dignified services [15]. Also policies to ensure the health workforce is available, accessible, acceptable and of high quality (AAAQ) contributes to expansion of effective coverage of health services [16]. The provision of acceptable, responsive and humane services without discrimination are critical to social inclusion in the health sector. Cultural competencies, no less important than clinical and public health competencies, help improve access to culturally acceptable services by socially excluded people and communities.

Societies need to dispel certain myths on social rights protection for migrants. Equitable social rights for migrants are not a ‘pull factor’ in destination countries; most migrants do not compare the benefits provided by welfare systems when choosing their destination countries [17]. Migrants are not a drain on national health systems but are tax-payers, in particular of consumption tax. Migrants are also workers who contribute to the economy of host countries, especially those facing labour shortages [18]. Thailand, which hosts 3.5 million migrants, of whom most are irregular migrants, has introduced migrant health insurance and implemented language and culturally friendly services [19].

Scientific communities have important roles to play; for example, developing a greater understanding on the causes of stereotyping, stigma and discrimination and devising innovative solutions [20]. More emphasis should be given to uncovering the inequalities and disparities across populations and understanding the mechanisms and complexities underpinning them.

Non-state actors can fill gaps; for example, the Culture Centre of the Deaf in Mongolia advocates awareness of UNCRPD and contributes to the CRPD shadow report. The Disabled People's International (DPI) Women's Network in Japan has addressed the multiple discrimination of women with disabilities; it also advocated for acting on synergies between the CRPD and the CEDAW in order to protect persons with multiple vulnerable attributes.

The whole society should have a responsibility to account and consider the rights of those who are vulnerable or excluded, and actions to reduce inequalities should be included in all actions towards promoting health.

## OVERARCHING SOLUTIONS

Universal health coverage (UHC) and other health related targets in the SDG are overarching policies in supporting social inclusion, and improving the level and distribution of health. Countries require a significant increase in government investment in strengthening primary health care–the close-to-client service that supports equitable access [21].

As social exclusion is dynamic and relational, generating research evidence and continuing monitoring in order to capture its dynamics will guide effective policy design to overcome these exclusion processes.

Regular monitoring and global reports by UN agencies and development partners contribute to awareness of progress or lack of progress, and hold state actors and other partners accountable to their commitments to various Conventions and other Political Declarations.

The ethical principle of leaving no one behind must be matched with triple government efforts to (a) strengthen health delivery systems and ensure services are equitably distributed, (b) progressively extend financial risk protection and (c) address the health needs of the vulnerable population who are not heard or counted, and who are excluded from economic prosperity. All these require responsive and accountable governments and the widespread engagement of active citizens who hold politicians and governments accountable. Social inclusion is often not about doing more, but rather about doing things differently to achieve a life with dignity for all.
